# The Aqueous Extract of Rhizome of *Gastrodia elata* Protected *Drosophila* and PC12 Cells against Beta-Amyloid-Induced Neurotoxicity

**DOI:** 10.1155/2013/516741

**Published:** 2013-09-23

**Authors:** Chun-Fai Ng, Chun-Hay Ko, Chi-Man Koon, Jia-Wen Xian, Ping-Chung Leung, Kwok-Pui Fung, Ho Yin Edwin Chan, Clara Bik-San Lau

**Affiliations:** ^1^Institute of Chinese Medicine, The Chinese University of Hong Kong, Shatin, New Territories, Hong Kong; ^2^State Key Laboratory of Phytochemistry & Plant Resources in West China, The Chinese University of Hong Kong, Shatin, New Territories, Hong Kong; ^3^School of Chinese Medicine, The Chinese University of Hong Kong, Shatin, New Territories, Hong Kong; ^4^School of Biomedical Sciences, The Chinese University of Hong Kong, Shatin, New Territories, Hong Kong; ^5^School of Life Sciences, The Chinese University of Hong Kong, Shatin, New Territories, Hong Kong

## Abstract

This study aims to investigate the neuroprotective effect of the rhizome of *Gastrodia elata* (GE) aqueous extract on beta-amyloid(A**β**)-induced toxicity *in vivo* and *in vitro*. Transgenic *Drosophila* mutants with A**β**-induced neurodegeneration in pan-neuron and ommatidia were used to determine the efficacy of GE. The antiapoptotic and antioxidative mechanisms of GE were also studied in A**β**-treated pheochromocytoma (PC12) cells. *In vivo* studies demonstrated that GE (5 mg/g *Drosophila* media)-treated *Drosophila* possessed a longer lifespan, better locomotor function, and less-degenerated ommatidia when compared with the A**β**-expressing control (all *P* < 0.05). *In vitro* studies illustrated that GE increased the cell viability of A**β**-treated PC12 cells in dose-dependent manner, probably through attenuation of A**β**-induced oxidative and apoptotic stress. GE also significantly upregulated the enzymatic activities of catalase, superoxide dismutase, and glutathione peroxidase, leading to the decrease of reactive oxidation species production and apoptotic marker caspase-3 activity. In conclusion, our current data presented the first evidence that the aqueous extract of GE was capable of reducing the A**β**-induced neurodegeneration in *Drosophila*, possibly through inhibition of apoptosis and reduction of oxidative stress. GE aqueous extract could be developed as a promising herbal agent for neuroprotection and novel adjuvant therapies for Alzheimer's disease.

## 1. Introduction

Beta-amyloid (A*β*) protein plays a central role in Alzheimer's disease (AD). Although the exact mechanism of the disease is unknown, the devastating effect of beta-amyloid is quite clear. The protein would self-aggregate into a plaque [1], which lead to the generation of reactive oxygen species, disruption of membrane potential, and increased vulnerability to excitotoxicity, and eventually cause neuronal death [2] and related cognitive defects [3]. Recent report postulated an increasing prevalence of dementia all over the world, from 36 million in 2010 to 66 million by 2030, with majority of AD [4]. Nowadays, AD threatens our aging population with the possible loss of memory and cognitive functions and leads to increasingly heavy health care burden to our future economy. Despite advances in medical interventions, Alzheimer's disease is fatal, and presently, there is no cure. Due to the complexity of pathology, AD is not very responsive to current western medications [5, 6]. Increasing attentions have turned to the conventional medicinal herbs, which are multitargeting, to search for a novel way of AD treatment [7, 8].

*Drosophila melanogaster* was recently developed as a model organism for drug/herbal screening for neurodegenerative diseases. It provides several unique features such as highly stable and fully-known genetics, highly conserved disease pathways, high-throughput, and very low comparative costs [9]. Most of the genes implicated in human AD pathogenesis have *Drosophila* homologs, including amyloid precursor protein (APP), *γ*-secretase, and tau [10]. However, there are some dissimilarities, such as the absence of *β*-secretase, which cause a defect in endogenous production of A*β*42 [11]. In this study, the *Drosophila* models that overexpress human A*β*42 would be used. The neurodegeneration would result in reduced lifespan, reduced locomotor activity, histological change to the neuronal structure, and eye degeneration [10, 12]. These pathological phenotypes could be observed within a few weeks, much faster than the development of these phenotypes in transgenic mice [13]. Therefore, application of *Drosophila* as model of AD provides excellent tools for performing drug/herb screens to identify small molecules/herbal formula that can suppress the toxicity associated with A*β* accumulation.

There is a long history of the use of medicinal herbs in the treatment of neurological disorders, like convulsion, stroke, and epilepsy, that is, *Poria cocos*, *Polygala tenuifolia*, *Uncaria rhynchophylla*, *Ginkgo biloba*, and *Lycium barbarum *[8, 14]. Modern pharmacological studies revealed that *Ginkgo biloba* possessed neuroprotective effects towards D-galactose [15], beta-amyloid [16], and ischemia-induced neuronal death [17]. *Uncaria rhynchophylla* also prevented D-galactose [18], beta-amyloid [19], 6-hydroxydopamine [20], and kainic acid-induced neurotoxicity [21]. Similar neuroprotective effects were found in other commonly used herbs in China [22–25]. Rhizome of *Gastrodia elata* (Tianma, GE) is also one of the commonly used traditional Chinese medicines. Many studies have been performed to evaluate the neuroprotective effects of GE and its biologically active ingredients against different kinds of neuronal damages. The nonpolar extract of GE inhibited the 1-Methyl-4-phenylpyridinium and glutamate-induced apoptosis in neuronal cells [26, 27]. Additionally, the nonpolar extract of GE protected mice and rat against kainic acid [28] and aluminum chloride-induced neuronal damages [29]. Its active ingredient, gastrodin, has been shown to possess a protective effect against hypoxia injury on neurons [30]. Other active compounds, hydroxybenzyl alcohol and vanillin, could ameliorate ischemic cerebral injury in rats [31], and prevent ischemic death of hippocampal neuronal in gerbils [32], respectively. Recently, an *in vitro* study indicated that the aqueous extract of GE enhanced proteolytic processing of APP towards the noncytotoxic nonamyloidogenic pathway [33]. Previously, studies revealed that APP processing affected the production of A*β*, which strongly correlated to the neuronal degeneration in AD pathology [34]. Mishra et al. demonstrated that that GE was able to inhibit *β*-site APP-cleaving enzyme 1 activity and promote *α*-secretase activity [33]. The inhibition of *β*-site APP-cleaving enzyme 1 reduces the cleavage of APP into A*β* [35], and the activation of *α*-secretase increases the cleavage of APP into soluble-APP-*α* [36, 37]. 

Although the nonpolar extract of GE was found to have various neuroprotective effects, extraction of GE with water is the traditional way of preparing Chinese medicine for human consumption. The active ingredient content of aqueous extract and nonpolar extract is theoretically different, which aqueous extract should have a higher content of hydrophilic gastrodin and polysaccharides and a lower content of less hydrophilic ingredients, such as hydroxybenzaldehyde, hydroxybenzyl alcohol, vanillin, and vanillyl alcohol. According to the Chinese pharmacopeia 2010, aqueous extract of GE is a traditional Chinese medicine that is widely used for treatment of convulsive disorders, headache, dizziness, and vertigo [38]. However, there is a lack of scientific evidence to support these medical claims. Based on these previous studies and the traditional use of GE, we hypothesize that aqueous extract of GE may also be effective in protecting neurons against beta-amyloid-induced neuronal death. Moreover, there is a lack of information relating to the *in vivo* neuroprotective effects of GE aqueous extract. In hope of finding an extract which could modulate APP cleavage and reduce neurotoxic effect from beta-amyloid, in the present study, we aimed to investigate the neuroprotective effects of GE on beta-amyloid-induced neurodegeneration in *Drosophila* and its related mechanisms using pheochromocytoma (PC12) cells. The mechanism of the neuroprotective effects of GE on the downstream pathway after the cleavage of APP to A*β* were studied, including the reactive oxygen species production and the activity of the antioxidative enzyme. The apoptosis caused by A*β* was also determined by propidium iodide (PI)/Annexin V staining and confirmed by caspase-3 activity assay.

## 2. Materials and Methods

### 2.1. Materials

A*β*_25–35_ peptide, 3-(4,5-dimethylthiazol-2-yl)-2,5-diphenyltetrazolium bromide (MTT), and caspase-3 assay kit were purchased from Sigma-Aldrich (St Louis, MO, USA). RPMI medium 1640, fetal bovine serum (FBS), horse serum (HS), and 2′,7′-dichlorodihydrofluorescein diacetate (H_2_DCFDA) were obtained from Invitrogen (Carlsbad, CA, USA). Annexin V-FITC and propidium iodide (PI) were obtained from BD Biosciences (San Jose, CA, USA). Superoxide dismutase and glutathione peroxidase assay kits were from Cayman Chemical (Ann Arbor, MI, USA). Catalase fluorometric detection kit was obtained from Enzo Life Sciences (Farmingdale, NY, USA). Formula 4–24 instant *Drosophila* medium was obtained from Carolina Biological Supply Company (Burlington, NC, USA).

### 2.2. Herbal Materials and Extraction

The raw herbs of the rhizome of *Gastrodia elata* were purchased from Chinese herbal stores in the Guangdong province in Mainland China. It was chemically authenticated using thin layer chromatography in accordance to the Chinese Pharmacopoeia 2010 and deposited in the museum of the Institute of Chinese Medicine, the Chinese University of Hong Kong, with voucher specimen number of 2010-3294. For extraction, the raw herbs Tianma were firstly washed with tap water to remove any contaminants. They were then cut into small pieces. The herbs were soaked with 10-fold of water (v/w) for 1 h, followed by extraction at 100°C for 1 h. Subsequent extractions were carried out with 10-fold of water (v/w) for another 1 h. The extracts were combined and concentrated under reduced pressure to give dry Tianma powdered extract. Ultimately, 48.90 g of the aqueous extract was obtained from 100.00 g of raw GE herb. The content of gastrodin was determined to be 2.2% w/w using high performance liquid chromatography, according to the method listed in the Chinese Pharmacopeia 2010 [38], which is higher than the requirement of 0.2% w/w.

### 2.3. *Drosophila* Strains

*Drosophila* strains used in this study were Oregon-R-C (OR) (#5), *w* ^*1118*^ (#3605), and *elav-GAL4* ^*C155*^ (#458) (Bloomington *Drosophila* Stock Center, Department of Biology, Indiana University, Bloomington, IN, USA). *UAS-A*β*42/CyO* and *GMR-A*β*42* ^*K52*^ *; GMR-A*β*42* ^*K53*^ heterozygous were gifts from Dr. M. Konsolaki (Rutgers University, USA). OR is a wild type *Drosophila*. *w* ^*1118*^ is a white-eye mutant with a deletion in the sex-linked white gene. *Elav-GAL4* ^*C155*^ is a mutant with an *embryonic lethal abnormal vision (elav)-GAL4* insert on the X chromosome. *UAS-A*β*42*/*CyO* is a mutant with an *UAS-A*β*42* insert and a *Curly of Oster (CyO)* balancer on the 2nd chromosome. *GMR-A*β*42* ^*K52*^; *GMR-A*β*42* ^*K53*^ heterozygous is a mutant with 2 copies of *Glass Multiple Reporter (GMR)-A*β*42 *inserts on the 3rd chromosome.

For longevity and climbing assay, genotypes of *Drosophila* used in this study were as follows: control: *elav-GAL4* ^*c155*^ */Y, A*β*42: elav-GAL4* ^*c155*^ */Y; UAS-A*β*42/+; +/+*. *Elav-GAL4* ^*C155*^ line was crossed with *w* ^*1118*^ line to produce control. *Elav-GAL4* ^*C155*^ line was crossed with *UAS-A*β*42/CyO* to produce *elav-GAL4* ^*c155*^ */Y; UAS-A*β*42/+; +/+*. The genotypes of newly hatched *Drosophila* are different between male and female. The genotype of the male offspring is *elav-GAL4* ^*c155*^ */Y; UAS-A*β*42/+; +/+, *while that of female offspring is *elav-GAL4* ^*c155*^ */w; UAS-A*β*42/+; +/+*. The existence of the wild type gene in par with our *elav-GAL4* ^*c155*^ promoter would half the overall expression of the transgene [39]. In order to minimize the error due to genetic difference, male was chosen in the present study. For the psuedopupil assay, *Drosophila* genotypes were as follows: Control: *OR*, A*β*42: *GMR- A*β*42* ^*K52*^ *; GMR- A*β*42* ^*K53*^ heterozygotes.

### 2.4. Effect of GE on Longevity of A*β* Expressing *Drosophila*

Genetic crosses were performed in the vials containing the diet with treatments. The normal control, which did not express A*β*, was maintained on the normal diet. The A*β* expressing control and the positive control were maintained on the normal diet and diet containing 10 mmol donepezil/g of *Drosophila* media, respectively, whereas the two GE groups were fed with diets containing 1 or 5 mg GE/g of *Drosophila* media, respectively. Newly hatched male *Drosophila* in each group was transferred to a new vial (30 *Drosophila* per vial), continued with their respective treatments, and incubated at 25°C. Dead *Drosophila* were counted on day 1 and 5 in a 7-day cycle, and the remaining live *Drosophila* were transferred to a new vial containing the same diet. The feeding lasted for 65 days. One hundred and fifty *Drosophila* were tested for each group.

### 2.5. Climbing Assay

Locomotor function of *Drosophila* was measured according to the climbing assay as previously reported by Lee et al. [40] with slight modifications. In brief, 30 male *Drosophila* were placed at the bottom of a 15 mL falcon tube and were given 10 s to climb up the tube. At the end of each trial, the number of *Drosophila* that climbed up to a vertical distance of 8 cm or above was recorded. *Drosophila* were tested on day 1 and 5 in a 7-day cycle. Each trial was performed three times. One hundred and fifty *Drosophila* were tested for each group.

### 2.6. Pseudopupil Assay

The control and A*β*42 *Drosophila* were treated with the same treatments as described above. *Drosophila* heads were examined under a light microscope (Olympus CX31; Olympus, Tokyo, Japan) as described previously [41]. Briefly, the compound eye of 5 days old *Drosophila* was viewed under microscope in a dark field. There were eight photoreceptors in each ommatidium, and seven of them were visible. Each photoreceptor projected a darkly staining rod, the rhabdomere, into the center of the ommatidium. Under the microscope, the rhabdomeres appeared as bright spots and rhabdomeres in each ommatidium were counted. In the control group, 7 rhabdomeres could be observed in each ommatidium. One hundred ommatidia were observed from 5 to 10 eyes, and the average rhabdomeres count per ommatidium was calculated. Three trials were conducted for each group.

### 2.7. Cell Culture and Drug Treatment

PC12 rat pheochromocytoma cells were obtained from American Type Culture Collection (Manassas, VA, USA) and maintained in RPMI medium 1640 supplemented with 10% (v/v) heat-inactivated HS and 5% (v/v) FBS at 37°C under 95% air/5% CO_2_. Cells were utilized for experiments during exponential growth. 

A*β*_25–35_ was dissolved in sterile distilled water at a concentration of 1.0 mM as a stock solution and preaggregated at 37°C for 7 days prior to use. Confluent cells were trypsinized, counted, and seeded on poly-L-lysine-coated 6-well culture plates at a density of 3 × 10^5^ cells/well and incubated for 24 h. After that, cells were treated with various concentrations of GE and 20 *μ*M of aggregated A*β*_25–35_ for 48 h.

### 2.8. Cell Viability Assay

Cell viability was determined using 3-(4,5-dimethylthiazol-2-yl)-2,5-diphenyltetrazolium (MTT) assay. Briefly, the cells were plated on poly-L-lysine-coated 96-well culture plates at the density of 1 × 10^4^ cells/well and incubated for 24 h. After that, the medium was replaced with fresh medium, and the cells were incubated with A*β*_25–35_ (1 *μ*M) in the presence or absence of aqueous extract of GE (250–1000 *μ*g/mL) for 48 h. Thereafter, cells were incubated with 30 *μ*L of MTT solution (final concentration, 1.5 mg/mL) for 4 h. The supernatant was then removed and 100 *μ*L of dimethyl sulfoxide was added to dissolve the formazan crystal. Plates were shaken for 10 min and optical density was determined with a microplate reader at 540 nm. The optical density of control cell was 100% viability.

### 2.9. Flow Cytometric Detection of Apoptosis

Apoptotic cells were quantified by Annexin V-FITC and PI staining by flow cytometry. Briefly, the treated cells were trypsinized and centrifuged at 450 ×g at 25°C for 5 min. The pellet was washed twice with ice cold PBS and resuspended with Annexin V binding buffer. Annexin V-FITC and PI were added according to manufacturer's instruction and incubated in dark at room temperature for 15 min. 300 *μ*L of binding buffer was added to each sample. The stained cells were analyzed by fluorescence-activated cell sorter (FACS). Ten thousands events were analyzed per sample.

### 2.10. Measurement of Apoptosis

The treated cells were collected and washed twice with ice cold PBS. PC12 cells were lysed in cell lysis buffer (50 mM Tris-HCl pH 7.4, 2 mM MgCl_2_, 0.1% Triton X-100) with 2 freeze/thaw cycles. The supernatant was collected after centrifugation at 15,000 ×g for 3 min; after that, the total protein concentration was determined by the bicinchoninic acid (BCA) assay, using bovine serum albumin (BSA) as a standard. The samples were then applied to caspase-3 activity assays, according to manufacturer's instructions. The activities were normalized using the total protein concentrations.

### 2.11. Measurement of Reactive Oxygen Species (ROS) Production

The 2,7-dichlorodihydrofluorescein diacetate (H_2_DCF-DA) method was used to measure intracellular ROS production. H_2_DCF-DA can pass through the cell membrane and oxidized by ROS to form the fluorochrome 2′,7′dichlorofluorescein (DCF). Therefore, H_2_DCF-DA was widely used to reflect the intracellular ROS content [42–44]. The treated cells were collected, washed twice with ice cold PBS, and incubated with H_2_DCF-DA (20 *μ*M) in the dark at 37°C for 15 min. Then cells were washed once with PBS and harvested for fluorescence-activated cell sorter (FACS) analysis. Ten thousands events were analyzed per sample.

### 2.12. Measurement of the Antioxidative Enzyme Activities

The treated cells were collected and washed twice with ice cold PBS. The cells were lysed in cell lysis buffer (50 mM Tris-HCl pH 7.4, 2 mM MgCl_2_, 0.1% Triton X-100) with 2 freeze/thaw cycles. The supernatant was collected after centrifugation at 14,000 ×g for 3 min, after that the total protein concentration was determined by the bicinchoninic acid (BCA) assay, using bovine serum albumin (BSA) as a standard. The samples were then applied to antioxidative enzyme activity assays, including glutathione peroxidase (GPx), superoxide dismutase (SOD), and catalase (CAT), according to manufacturer's instructions. The activities were normalized using the total protein concentrations.

### 2.13. Statistical Analysis

Multiple group comparisons were performed using one-way analysis of variance (ANOVA) followed by Dunnett's test to detect intergroup differences. Comparisons for survival assay were performed using Log-Rank analysis and chi-square comparison.

All statistical analyses were performed using GraphPad Prism version 5.0 for Windows (GraphPad Software Inc., California, USA). The data were expressed as mean ± standard deviation (SD). A value of *P* < 0.05 was considered statistically significant.

## 3. Results

### 3.1. Tianma Prolonged the Lifespan and Improved Locomotor Abilities of A*β*-Expressing *Drosophila*

**Figure 1:**
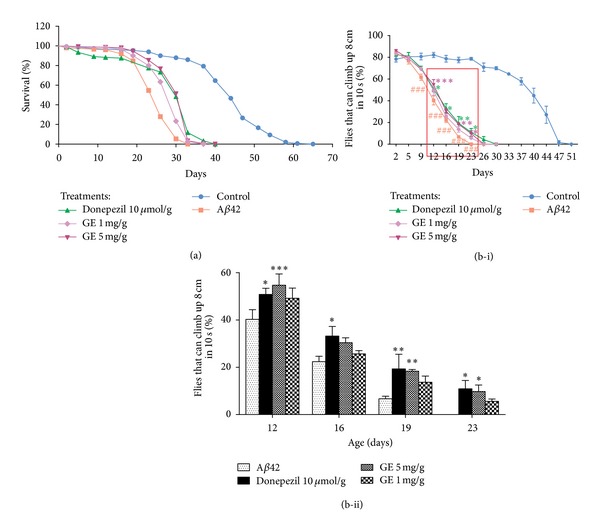
Intake of GE increases the (a) lifespan and (b-i) locomotor activity of A*β*-expressing *Drosophila*. The lifespan of A*β*42 group (squares in red) is shorter than the control group (circles in blue), while GE (asterisks and triangles in purple) or donepezil (triangles in green) treatments delay the mortality of the *Drosophila*. (b-ii) is an amplification of the region from days 12 to 23 showing the differences among the A*β*42 group and the treatment groups. The percentage of *Drosophila* climbing up 8 cm in 10 seconds was increased by GE or donepezil treatments when compared with A*β*42 group. Results are the means ± SEM from five independent crosses. ^###^ *P* < 0.001 relative to control; *P < 0.05, **P < 0.01, ***P < 0.001 relative to A*β*42 *Drosophila* by one-way ANOVA for locomotor activity. Log-Rank analysis and chi-square comparison were applied to the survival data and *P* < 0.001 was obtained when comparing A*β*42 *Drosophila* and Donepezil 10 *μ*mol/g or GE 1 mg/g or 5 mg/g treated ones (*n* = 150).

In the present study, we evaluated the neuroprotective effect of aqueous extract of GE, using *Drosophila* AD model. Before performing the experiments, we evaluated the effect of 5 and 50 mg GE extract/g of *Drosophila* media on food intake of *Drosophila*. Both GE treatments did not affect the food intake of *Drosophila *(data not shown), which ensured no experimental differences were due to the alteration of feeding behavior. For lifespan experiment, A*β*42 *Drosophila* showed a reduction of median and maximum lifespan by 17 days and 32 days when compared with control, respectively. Both GE treatments significantly improved the survival of *Drosophila* (Figure 1(a)). At 1 mg GE extract/g of *Drosophila* media, median and maximum lifespan were increased by 4 days (12.0%) and 4 days, respectively (*P* < 0.001 for mean increases). At 5 mg GE extract/g of *Drosophila* media, median and maximum lifespan were increased by 7 days (26.9%) and 7 days, respectively (*P* < 0.001 for mean increases).

For locomotor abilities determination, A*β*42 *Drosophila* showed significant impaired locomotion from age of day 9 onwards (Figure 1(b-i)). GE-treated flies showed an improvement in locomotor activity from age of days 12 to 23. At day 12, 19, and 23, 5 mg GE extract/g of *Drosophila* media resulted in a 14.4%, 11.6%, and 9.74% improvement in locomotion, respectively (*P* < 0.001, *P* < 0.01, *P* < 0.05) (Figure 1(b-ii)) when compared with the A*β*42 *Drosophila *without GE treatment.

### 3.2. Tianma Rescued Neurodegeneration in Ommatidia of A*β*-Expressing *Drosophila*

**Figure 2:**
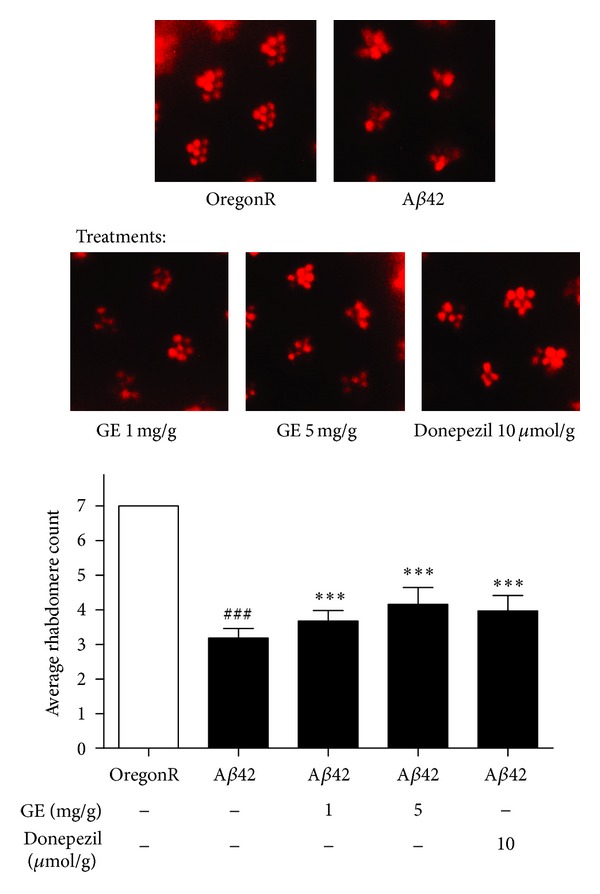
Rhabdomere count in the pseudopupil assay. Regular array of 7 ommatidia (bright red spots) was observed in OregonR eyes. Degeneration of ommatidia was observed in the A*β*42 group, while the degeneration is improved by GE or donepezil treatments. ^###^ *P* < 0.001 relative to OregonR; ***P < 0.001 relative to A*β*42 *Drosophila* with no treatment by one-way ANOVA. Results are the means ± SEM from 3 independent crosses. One hundred ommatidia were observed from 10 eyes of 5 *Drosophila *from each group in each trial.

We analyzed the effect of A*β*42 on degeneration of retinal tissue of *Drosophila*, which were mainly neurons. A*β*42 *Drosophila* contained significantly more degenerating rhabdomeres, compared with OregonR. The number of degenerated rhabdomeres was 3.82 ± 0.09. A*β*42 *Drosophila* treated with GE (1 and 5 mg/g of *Drosophila* media) had significantly rescued rhabdomere in each ommatidium, with an increase of 0.49 and 0.97 rhabdomere count per ommatidium, respectively (Figure 2), which reflected a preventive effect of GE on neurodegeneration. The preventive effect was comparable to donepezil medication (10 *μ*mol/g of *Drosophila* media), in which there was an increase of 0.78 rhabdomere count per ommatidium than the A*β*42 *Drosophila*.

### 3.3. Tianma Reduced A*β*-Induced Cytotoxicity in PC12 Cells and Prevented A*β*-Induced Apoptosis

**Figure 3:**
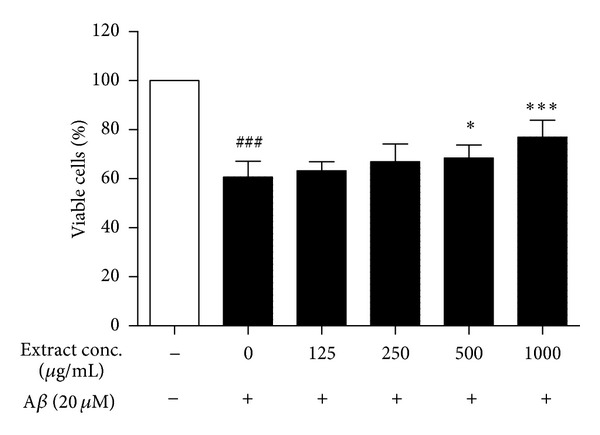
Protective effect of GE on A*β*-induced cytotoxicity in PC12 cells. Effect of 48 h treatment of GE extract on the viability of PC12 cells was determined by MTT assay. Results are the means ± SD from three separate experiments. ^###^ *P* < 0.001 relative to control; *P < 0.05, ***P < 0.001 relative to A*β* treatment only by one-way ANOVA.

**Figure 4:**
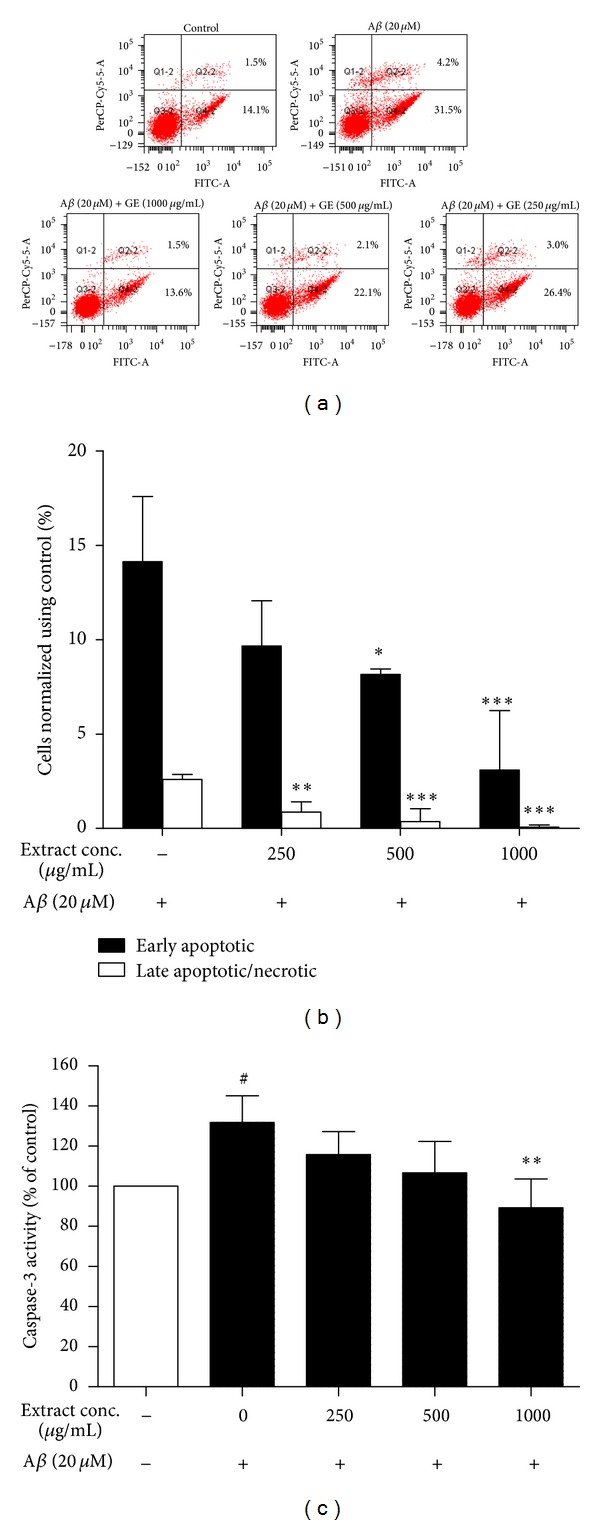
Antiapoptotic effect of GE on A*β*-induced cytotoxicity in PC12 cells. (a) Representative plots for the flow cytometric analysis. (b) GE extract reduced A*β*-induced apoptosis in flow cytometric analysis. The fluorescence intensity was measured after PC12 cells were exposed to 20 *μ*M A*β* for 48 h, followed by incubation with Annexin V-FITC and PI for 15 min. (c) 48 h treatment of GE extract attenuated A*β*-induced activation of caspase-3. Results are the means ± SD from three separate experiments. ^#^ *P* < 0.05 relative to control; *P < 0.05, **P < 0.01, ***P < 0.001 relative to A*β* treatment only by one-way ANOVA.

Exposure of PC12 cells to aggregated A*β*_25–35_ (20 *μ*M) for 48 h caused significant cytotoxicity. Concentration of GE in the range from 125 to 1000 *μ*g/mL was identified to be non-toxic to PC12 cells by MTT assay (data not shown). The high concentration of GE used is also correlated to its high extraction yield in water (48.9%), compared with the yield of less than a few percent in extraction by nonpolar solvents. Our results demonstrated that GE imposed significant protective effect against A*β*_25–35_-induced damage in a dose dependent manner, with the maximum effect observed at 1000 *μ*g/mL (Figure 3). Therefore, concentration of GE in the range from 250 to 1000 *μ*g/mL was selected for the further apoptosis study. In this regard, we investigated the effect of GE on A*β*_25–35_-induced apoptosis using Annexin V-FITC and PI staining. Early apoptotic (PI: negative, Annexin V: positive) cells and late apoptotic (PI and Annexin V: positive) cells were quantified by flow cytometry. For the control group treated with A*β*_25–35_ only, the normalized percentages of early and late apoptosis induced by A*β*_25–35_ were 14.1 ± 3.5% and 2.6 ± 0.3%, respectively. For the treatment groups, the percentage of early and late apoptosis induced by A*β*_25–35_ with treatment of GE were 9.7 ± 2.4% and 0.9 ± 0.6% for 250 *μ*g/mL, 8.2 ± 0.3% and 0.4 ± 0.7% for 500 *μ*g/mL, and 3.1 ± 3.1% and 0.1 ± 0.1% for 1000 *μ*g/mL (Figure 4(b)). The results suggested that GE can reduce A*β*_25–35_-induced apoptosis dose dependently. To further confirm the antiapoptotic effects of GE against A*β*_25–35_-induced toxicity, the activity of crucial mediator of apoptosis caspase-3 was assessed. Caspase-3 activity was increased by 31.8 ± 13.4% with A*β*_25–35_ treatment, and the increase in activity was attenuated dose dependently with treatment of GE (Figure 4(c)). At 1000 *μ*g/mL of GE, the caspase-3 activation was totally abolished and reverted to the normal activity level of the PC12 cells without A*β*_25–35_ treatment.

### 3.4. Tianma Prevented A*β*-Induced Oxidative Stress

**Figure 5:**
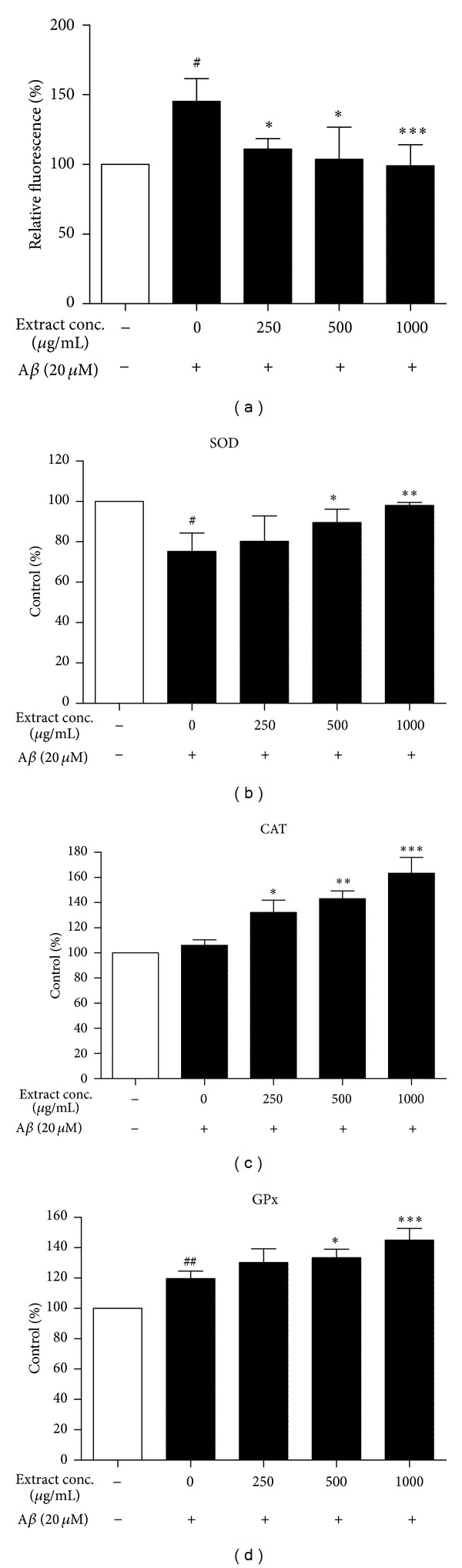
Antioxidative effect of GE on A*β*-induced cytotoxicity in PC12 cells. (a) GE extract reduced A*β*-induced oxidative stress in flow cytometric analysis of DCF positive cells. The fluorescence intensity of DCF was measured after PC12 cells were exposed to 20 *μ*M A*β* for 48 h, followed by 20 *μ*M H2DCF-DA for 15 min. 48 h treatment of GE extract increased the activities of antioxidative enzymes (b) superoxide dismutase (c) catalase and (d) glutathione peroxidase in 20 *μ*M A*β*-treated cells. Results are the means ± SD from three separate experiments. ^#^ *P* < 0.05, ^##^ *P* < 0.01 relative to control; *P < 0.05, **P < 0.01, ***P < 0.001 relative to A*β* treatment only by one-way ANOVA.

Figure 5(a) shows that 20 *μ*M A*β*_25–35_ elevated the production of ROS from 100% to 145.2 ± 16.3%, whereas the fluorescence intensity in GE-treated groups decreased significantly (110.9 ± 7.5%, 103.7 ± 23.1%, and 99.0 ± 15.1%, resp.). The decrease of fluorescence by GE reflected the reduction of ROS content, which possibly caused by the activation of antioxidative enzymes.

The activities of antioxidative enzymes (SOD, CAT, and GPx) in untreated PC12 cells and in those treated with 20 *μ*M A*β*_25–35_ alone or with GE together are presented in Figures 5(b)–5(d). Activity of SOD was decreased by 24.75 ± 9.07% in the cells exposed to 20 *μ*M A*β*_25–35_ (Figure 5(b)). Exposure to 20 *μ*M A*β*_25–35_ did not significantly affect the activity of CAT (Figure 5(c)) and induced a 19.70 ± 4.87% increase in activity of GPx (Figure 5(d)). With 1000 *μ*g/mL of GE treatment, the activity of SOD was reverted to the normal activity level of the PC12 cells without A*β*_25–35_ treatment, while the activity of CAT was enhanced by 63.30 ± 12.58% compared with the normal control. Moreover, 1000 *μ*g/mL of GE further increase the activity of GPx to 45.00 ± 7.71% higher than the normal control. Overall, treatment with different doses of GE significantly and dose-dependently enhanced the activities of SOD, CAT, and GPx (Figures 5(b)–5(d)).

## 4. Discussion

In the present study, we have presented the first evidence that the aqueous extract of GE could significantly ameliorate the adverse morphological changes from A*β* protein in *Drosophila*, as indicated by improving locomotor abilities, prolonging the lifespan, and rescuing neurodegeneration in ommatidia in A*β*-expressing *Drosophila*. *In vitro* experiments showed that A*β*-treated cultures exhibited characteristic features of ROS production, apoptosis, and cell death in PC12 cells. GE aqueous extract attenuated A*β*-induced cytotoxicity effectively, probably through increasing the activities of antioxidative enzymes so as to reduce overall oxidative stress and subsequently inhibiting A*β*-induced apoptosis.

Two *Drosophila* lines were used to overexpress different levels of A*β*, one was using *GMR* promoter and one was using *GAL4-UAS* system. *GMR* promoter element directs the expression of the protein at the eye imaginal disc. The advantage of expressing only in the eye is that flies producing a highly toxic protein may still be viable. Rapid and severe degeneration of the ommatidia (eyes of *Drosophila*) was achieved due to the presence of two copies of gene encoding for A*β* in our *Drosophila* with *GMR* promoter [10]. For the latter one, the *GAL4-UAS* system is more complex. Tissue-specific expression of the *UAS-A*β*42* is achieved by crossing the transgenic *Drosophila* with driver lines that control tissue-specific expression *GAL4*, which would bind with *UAS* to activate gene transcription. *Elav-GAL4* is a commonly used pan-neuronal driver that directs the expression of transgene throughout the brain, neuronal system, and retina of the *Drosophila* [45]. The advantage of this model is that the lethal gene can be carried in the parents without affecting their viability and fecundity. In this study, *UAS-A*β*42* would be crossed with e*lav* ^*C155*^ *-GAL4* to express A*β*42 in the brain and the whole neuronal system and gradually accumulate to induce the degenerative phenotypes, such as the pathological morphologies and behavioral changes, in weeks. Therefore, to identify the efficacy against A*β* toxicity, the rationale of the current assays aimed to see whether GE aqueous extract can rescue retinal degeneration, locomotion and climbing deficits, and increase the lifespan of the flies, restoration of normal activity.

Using these two *Drosophila* models, the *in vivo* effects of GE aqueous extract in Alzheimer's disease were studied. Firstly, we found that GE aqueous extract reduced the neurotoxic effect of A*β* to ommatidia. The degree of degeneration of ommatidia reflected the extent of neurodegeneration [46], based on the fact that photoreceptors were neurons in nature. Overexpression of A*β* causes plaque formation and neuronal degeneration, which was responsible for the eye morphological changes [10, 12]. The intake of GE extract reduced the adverse effect of A*β*-associated plaque formation and rescued the eye phenotype. Similar findings were observed in the other *Drosophila *model with systemic pan-neuronal A*β*42 expression. GE aqueous extract significantly prolonged the lifespan and improved locomotor dysfunction of the flies. We also found that the beneficial effects of GE were comparable with the medicine donepezil. Hong et al. recently reported that Chinese traditional medicinal prescription SuHeXiang Wan improved the longevity and locomotor ability using the same *Drosophila *model system [47]. The results of the current study suggested that GE aqueous extract confers a therapeutic potential to AD-like pathology of A*β*42 overexpressing in different *Drosophila* models. In our *Drosophila* model, the AD-like pathology was caused by the neurotoxic A*β* produced, secreted to and aggregated in the extracellular matrix [48], yet there was no previous report on the effect of A*β* on apoptosis and oxidative stress on *Drosophila*. Previous reports revealed the direct proportional relationship between the manifestations of neuronal dysfunction in *Drosophila*, such as locomotor deficits and reduced lifespan, and aggregation rate of the A*β*, which provide evidence that the aggregated A*β* is the primary determinant of the pathological behavior in the *Drosophila* system [49]. To study pathogenic mechanisms, we have developed *in vitro* model that recapitulate many of the signature events in A*β* neurotoxicity including the accumulation extracellular aggregated A*β*, leading to apoptotic events and formation of reactive oxygen species. Basing on previous studies that were using PC12 cells as a platform to express and study the action mechanisms of *Drosophila* proteins [50], we postulate that the PC12 cells would be able to mimic the cellular environment of the *Drosophila*. Moreover, there was a well-established platform using PC12 cells and *Drosophila* toscreen and validate aggregation inhibitors of polyglutamine, which resulted in neurodegeneration [51, 52]. The platform suggested that PC12 cells and *Drosophila* would have correlation in neurodegeneration mechanisms. Therefore, PC12 cell line was used to explain the* in vivo* effects in the present study.

For the *in vitro* mechanistic studies, PC12 cell line, which is originated from transplantable rat adrenal pheochromocytoma, was used. Due to their similarity with sympathetic neurons and their reversible differentiation response to nerve growth factor [53], PC12 cells were widely used in the study of neuronal differentiation [54], neuronal function [55], and neurodegeneration [56, 57]. A*β*-induced cytotoxicity on PC12 cell line is widely used to study the AD-related neurodegeneration [58]. In the present study, we adopted this cell line and found that GE possessed protective effect against A*β*-induced cell death in MTT assay. Previously, Kim et al. had also demonstrated that the ethyl ether fraction of GE was able to protect A*β*-induced IMR-32 neuroblastoma cell death [59]. However, the content of active ingredients in the ethyl ether extract was expected to be different from the aqueous extract. Although the dose of extract used in the study was as low as 10 *μ*g/mL, the extraction yield of the extract was only 1.12%. When comparing with the present study of extraction yield of 48.90%, the dose was equivalent to 420.61 *μ*g/mL in the present study, which is similar to the present dose of 500 *μ*g/mL. Moreover, the study only demonstrated the protective effect using MTT assay, but lacked further elucidation of any protective mechanisms. A complete picture from the *in vivo *effect to the downstream neuroprotective mechanisms was yet to be provided, and the present study was the novel one targeting this. Extensive evidence shows that neuron cell death in AD is mediated by apoptosis [60, 61]. For instance, postmortem analysis of AD brain shows that there is DNA fragmentation in neurons and glia of hippocampus and cortex as detected by TdT-mediated dUTP nick end labeling [62]. It was also found that the extracellular accumulation of A*β*, which triggers the intracellular formation of neurofibrillary tangles [63], leads to the loss of cholinergic neurons [64]. Hence, a common theory believed that the pathological neuronal loss in AD is through apoptosis, which may be caused by A*β* accumulation and cytotoxicity [2, 34]. In order to elucidate the possible mechanisms of the neuroprotective effect, the antiapoptotic effects of GE were determined by flow cytometry using PI/Annexin V staining method and caspase-3 activity assay. Our PI/Annexin V data demonstrated that GE could strongly attenuate not only the early stage but also the late stage of apoptosis/necrosis induced by A*β*. Besides, we also found that GE could suppress A*β*-induced caspase-3 activity, which provided further evidence in antiapoptosis.

Although the exact underlying mechanism leading to A*β*-induced apoptosis was not well understood, oxidative stress caused by the A*β* plaque was widely believed to seriously impair various cellular function and play an important role in apoptosis [65, 66]. Therefore, reducing reactive oxygen species (ROS) production was a promising approach to inhibit A*β*-induced apoptosis. It has been previously reported that the nonpolar fractions of GE and its active constituents could inhibit ROS generation [32, 67]. In this study, we found that the aqueous extract of GE also possessed strong antioxidative action, which decreased the H_2_DCF-DA-labeled ROS accumulation in PC12 cells. Antioxidative action can be mediated by 2 mechanisms: activation of antioxidative enzymes and direct free radical scavenging [68]. Antioxidative enzymes, including superoxide dismutase and catalase, convert superoxides, a strong ROS, to hydrogen peroxide and then to water. Glutathione peroxidase catalyzes the reaction of glutathione and hydrogen peroxide, which is a crucial endogenous antioxidative mechanism, to water [69]. In the present study, although A*β* did not affect the activity of CAT, the impairment of the upstream SOD would cause the accumulation of superoxides. On the other hand, the upregulation of GPx by A*β* was possibly a response to the increased ROS and facilitated the action of glutathione. Due to the fact that CAT and GPx could not breakdown superoxides, the accumulation of superoxides may be the explanation for the observed oxidative stress after the A*β* treatment. Our results also demonstrated that GE up-regulated the activity of SOD, CAT, and GPx during A*β*-insult. The activity of SOD was retained, which resume the breakdown of superoxides to hydrogen peroxide. The up-regulation of CAT and GPx can promote the clearance of ROS, and that partially explained the antioxidative action for GE. Other studies also demonstrated that both nonpolar and polar fractions of GE have hydroxyl radical scavenging activity and reduce lipid peroxidation [67, 70, 71]. Its active constituents, including vanillyl alcohol, vanillin, hydroxybenzyl alcohol, and hydroxybenzaldehyde, were found to be potent antioxidants [32, 72]. These compounds can be found in aqueous extract of GE [73, 74].

As aqueous extract of GE was widely and traditionally used in Chinese medicine as a supplement in diet and an herbal medicine [75], and the further development of GE as novel non-toxic preventive/treatment interventions for life-threatening neurodegenerative diseases, such as AD, is possible. In order to confirm our current findings, further investigation of the neuroprotective effect of GE to mammalian AD model is necessary. Since the traditional way of consuming Chinese herbs is to be taken orally, the gastrointestinal metabolic ingredients of GE are the final effective elements. However, very limited information were found regarding the pharmacokinetic data of GE aqueous extract, except that gastrodin was known to be metabolized to *p*-hydroxybenzyl alcohol [76], and both gastrodin and *p*-hydroxybenzyl alcohol possess significant free radical scavenging and memory consolidation effects [77, 78]. Hence, further pharmacokinetic studies are required to understand the post metabolism ingredients of GE. Besides, further investigation is needed to determine the clinical efficacy and safety of GE in human subjects because the presence of blood-brain barrier (BBB) may block those beneficial active ingredients from the brain. Although BBB exists in *Drosophila* and serves the function of blocking the passage of ions and small molecules [79, 80], the *Drosophila's* BBB is morphologically different from the mammalian one [81, 82]. Nevertheless, some previous studies demonstrated that intravenous administration of gastrodin and hydroxybenzyl alcohol were able to pass through BBB in rats [83, 84]. However, the pharmacokinetics of GE in the human brain is yet to be investigated.

## 5. Conclusions

In conclusion, the present study demonstrated the novel use of aqueous extract of GE against A*β*-induced neurodegeneration in *Drosophila*. Its effect is mediated through the increasing activity of antioxidative enzymes and reducing oxidative stress in cells, together with the inhibition of caspase-3, leading to the attenuation of apoptosis. Based on these findings, we suggest developing GE aqueous extract as a potential therapeutic intervention for neurodegenerative diseases, such as Alzheimer's disease.
